# Sonographic and Clinical Features of Typhlitis in Pediatric Cancer Patients on Chemotheaphy at Tikur Anbessa Specialized Hospital, Ethiopia, 2021

**DOI:** 10.4314/ejhs.v32i1.5S

**Published:** 2022-10

**Authors:** Hansel J Otero, Sissay Eshetu, Daniel Zewdneh, Daniel Hailu, Yocabel Gorfu, Hermon Miliard Derbew

**Affiliations:** 1 Department of Radiology, Faculty of Medical Science, Institute of Health, Jimma University, Ethiopia; 1 Department of Pediatric Radiology, Children's Hospital of Philadelphia (CHOP), Perlman School of Medicine, Pennsylvania University, USA; 2 Department of Radiology, Faculty of Medical Science, Institute of Health, Addis Ababa University, Ethiopia; 3 Department of Pediatrics and Child Health, Faculty of Medical Science, Institute of Health, Addis Ababa University, Ethiopia

**Keywords:** Typhlitis, chemotherapy, pediatric malignancy, ultrasound imaging

## Abstract

**Background:**

Typhlitis, (neutropenic enterocolitis), is a necrotizing enteropathy of the right colon, and is characterized by the clinical triad of fever, abdominal pain, neutropenia and imaging findings of right-side colonic inflammation. It is seen in the setting of severe neutropenia in immune suppressed patients who undergo treatment for malignancies, in those who have organ transplant(s) or congenital or other acquired immunosuppression. We report the clinical and imaging findings of typhlitis in pediatric cancer patients who had received chemotherapy in the largest tertiary center in Addis Ababa, Ethiopia over a period of 20 months.

**Methods:**

The medical records of hospitalized cancer patients on treatment and with suspected typhlitis and with ultrasound reports were screened (November 2018- July 2020). Retrospective analysis of the clinical and sonographic data of those with typhlitis was done.

**Results:**

Typhlitis was identified in 4.2% (12/286) of the patients on chemotherapy. 11 (91.7%) had hematologic malignancies (leukemia, lymphoma), one had a solid tumor (Head and neck embryonal RMS). Most (83.3%) had abdominal pain, diarrhea and neutropenia. Fever was identified in 67.7%. All had ultrasound evidence of typhlitis. and treated with IV antibiotics. Neither complications requiring surgical intervention nor death were seen.

**Conclusion:**

The magnitude of disease was comparable to what had previously been reported in other studies. While the presence of clinical a triad should prompt suspicion for the diagnosis, sonography can be used for confirmation and follow up obviating radiation, with good access in a resource limited setting

## Introduction

Typhlitis, also known as neutropenic enterocolitis, is a necrotizing enteropathy of the right colon (1). It is most often seen in the setting of severe neutropenia in immune-suppressed patients who undergo organ transplantation or treatment for malignancies as well as in those with congenital or acquired causes of immune-suppression (1). It is characterized by fever and abdominal pain in a neutropenic patient (2). On imaging, it is characterized by bowel wall thickening (0.3 cm or greater), peri colonic fluid and inflammation of the peri colonic fat usually isolated to the cecum, adjacent terminal ileum and ascending colon with left colonic involvement being uncommon, but the definitive diagnosis is made histologically (1, 2, 3, 4). Common complications are sepsis, obstruction and abscess formation (5). There is no optimal agreed upon management for patients with typhlitis but most receive conservative medical management consisting of a combination of bowel rest, total parenteral nutrition and use of broad-spectrum antibiotics. The use of granulocyte colony stimulating is also mentioned while surgery is reserved for those who have evidence of bowel perforation or abscess formation (2,4,5,6).

Recent studies showed overall incidences of lower than 10% in patients on chemotherapy (2,4,10). However, there is a paucity of data on the incidence of typhlitis in the setting of low to middle income countries (LMIC). Those available were done in reference to patients with acute leukemia. In a study done in Egypt, the incidence of typhlitis was found to be 24% (49/203) with all patients requiring ICU admissions resulting in a high 30-day mortality rate of 44.8% (11). A separate retrospective evaluation of 75 pediatric patients with acute leukemia in Turkey showed the incidence of typhlitis to be 4.5% with a high mortality rate of 20% (12). Although the recorded incidences in these two studies appear to be in the range of incidences in high income counties, the mortality rates were markedly higher. These could be attributed to poverty-related factors such as low parental literacy, suboptimal living conditions, and distance from healthcare facilities (13).

This study aims to correlate demographic, clinical and laboratory data with sonographic imaging findings of right colonic wall changes to determine the overall clinical and imaging pattern of the disease in pediatric cancer patients who had undergone chemotherapy.

## Material and Methods

This was a cross-sectional retrospective study of pediatric haemato-oncology patients in our institution, a public tertiary hospital in Ethiopia that is the only facility providing chemotherapy for the treatment of pediatric cancer in the country. The study was approved by the Research and Ethics Committee.

Children on chemotherapy with a clinical diagnosis of typhlitis were screened between November 2018 and July 2020. For the purpose of this study, typhlitis was defined as the presence of neutropenia, with at least one clinical symptom or sign (i.e. abdominal pain or fever) and confirmatory imaging signs (thickened colonic wall >0.3cm) on ultrasound scan. (The diagnosis of typhlitis is possible in such patients as long as the other clinical and imaging findings are present) (4). Neutropenia was defined as an ANC (absolute neutrophil count) of <500 cells/mm^3^ or an ANC that is expected to decrease to <500 cells/mm^3^ during the next 48 h (14). Fever was defined as a single oral temperature ≥ 38.3°C or a single axillary temperature of ≥ 37.8°C (14). Patients aged >15yr, those not on chemotherapy and those with unconfirmed malignancy were excluded.

A non-probability convenience sampling method was employed. 286 children fulfilling the above inclusion criteria were included in the 20-month study period. Data, including those from imaging reports, were reviewed and collected using a structured questionnaire and analyzed with nonparametric statistical methods using SPSS software package. A confidence level of 95% with confidence interval (CI)of 0.05 was used.

***Image acquisition and interpretation:*** Ultrasound studies were performed using a Sonoscape SSI-800 TM ultrasound machine with a L751 10-5MHz TM, linear array high frequency probe and a 2P1 4-2MHzTM, phased sector array probe. The procedure was performed by senior residents and supervised by the consultant pediatric radiologist before reporting. The protocol included scanning in both transverse and sagittal planes along the entire length of the bowel with compression anteriorly and posteriorly. Thickness of bowel loops involved was recorded. Peritoneal fluid was also assessed during the exam.

***Clinical outcomes***: We recorded the final disposition of the patient according to the records into discharge, cured, or dead.

## Results

**Figure 1:**
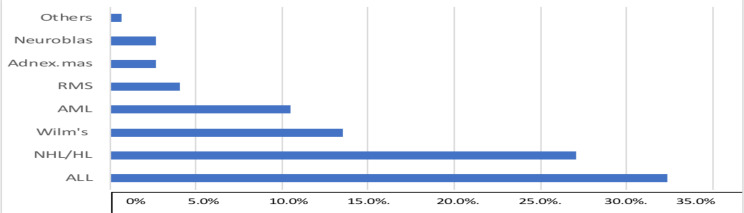
Disease distribution at admission. (RMS): rhabdomyosarcoma. AML (acute myeloid leukemia), NHL/HL (Non-Hodgkin's and Hodgkin's lymphoma).

Of the 286 patients, 174(60.8%) were boys and 112 (39.2%) were girls. The mean age at presentation was 6 years (range: 3months-13 years). The most commonly diagnosed malignancies were hematologic ones (leukemias and lymphomas) constituting about 70.3% of cases (figure 1).

Twelve patients were diagnosed with typhlitis; 10 (83.3%) were male and 2 (16.7%) were female. The mean age at diagnosis was 7.7 years (Range: 3–12 years). Five patients had non-Hodgkin lymphoma (NHL), 4 patients had ALL (acute lymphoblastic leukemia), 2 patients had AML (acute myeloid leukemia) and 1 patient had embryonal rhabdomyosarcoma (RMS).

Eleven of the patients (91.7%) had their diagnosis confirmed histologically. One patient with mediastinal NHL had no tissue diagnosis and was diagnosed on imaging alone (via contrast enhanced chest CT) (Table 1).

**Table 1:** Disease distribution of patients with typhlitis

Disease	Frequency (%)	Cumulative percent
NHL-Intraabdominal	4 (33.3)	33.3
HR-ALL	3 (25.0)	58.3
AML-M2	1 (8.3)	66.7
AML-M3	1 (8.3)	75.0
SR-ALL	1 (8.3)	83.3
NHL-Mediastinal	1 (8.3)	91.7
RMS	1 (8.3)	100
Total	12 (100)	

The commonest signs and symptoms associated with typhlitis were abdominal pain and diarrhea seen in 83.3 % of patients followed by fever, vomiting and abdominal tenderness (Table 2).

**Table 2:** Signs and symptoms of patients with typhlitis

Sign/symptom	Percent
Abdominal pain	83.3
Diarrhea	83.3
Fever	66.7
Vomiting	50.0
Abdominal tenderness	41.7
Nausea, constipation, bleeding per rectum	0

Neutropenia was recorded in 10 (83.3 %) patients. Two patients (16.7 %) had typhlitis in the absence of neutropenia. The overall mean absolute neutrophil count (ANC) was 288.3 cells/mm3 with maximum of 1900 and minimum of 0 cells/mm3 respectively (Table 3).

**Table 3:** Absolute neutrophil count (**ANC**) of patients

ANC	Number (%)
Neutropenia (ANC<500 cells/mm3)	10 (83.3)
Profound neutropenia (ANC <100cells/mm3)	8 (80)
No neutropenia	2 (16.7)
Total	12 (100)

Twenty five percent of the patients were on HR-ALL 4-drug induction protocol, while 25% were on NHL ALCL A2 phase and the rest comprising 8.3% each were on various treatment regimens according to their primary diagnosis.

Of the patients who developed typhiltits, 7 (58.3 %) were on chemotherapy when they developed symptoms. These were all of the 4 patients with ALL on induction phase, 1 patient each with AML(M3), mediastinal NHL and embryonal RMS.

**Figure 2:**
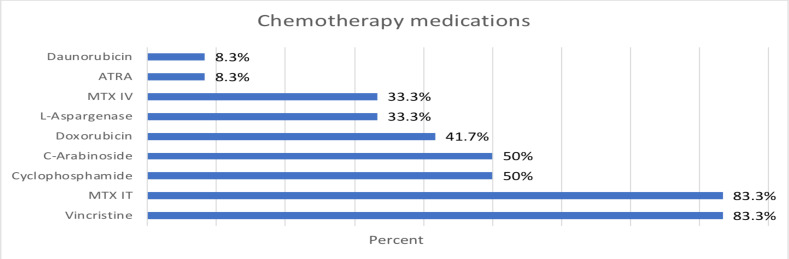
Bar chart of types of chemotherapy drugs taken in percent.

Vincristine (Intravenous) was part of the chemotherapy regimen in 83.3% of patients, followed by intrathecal methotrexate being used in 75%. Cyclophosphamide and cytosine arabinoside were used in 50% (Figure 2).

Ten patients (83.3%) had received steroid as part of their chemotherapy regimen either in the form of dexamethasone or prednisolone. One of the two patients who had not received steroid as part of chemotherapy (patient with AML) was treated with IV dexamethasone for management of elevated intracranial pressure.

**Figure 3:**
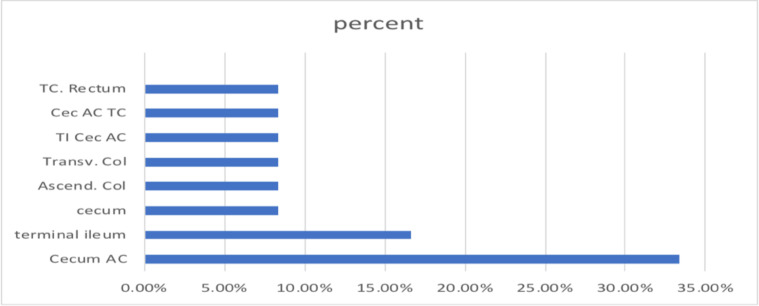
Bar chart of bowel segments involved on ultrasound in percent.

With regards to treatment, all patients were on IV antibiotics. The most commonly used being IV metronidazole in 58.3% followed by ceftriaxone and vancomycin in 50% of patients each. The least used medications were ciprofloxacin and cloxacillin (8.3% or 1 patient each). The antifungal fluconazole and antiviral acyclovir were used in 25% of patients. Amphotericin B was given to only one patient (8.3%) (Figure 3). None of the patients underwent surgery.

***Ultrasound findings:*** The commonest involved sites identified were the cecum and ascending colon in 4 patients (33.3%) followed by involvement of the terminal ileum only in 2 (16.7%) (Figure 3). The mean reported thickness of involved bowel loops was 11.1mm (Range 5–40mm) (n=11).

Mesenteric fat stranding was mentioned in 1 patient each. Peritoneal fluid collection without full descriptive factors (site, volume, echogenicity, and complexity) was mentioned in one third of cases. The rest of the reports did not comment on the presence or absence of fluid collection.

Terminal illeal distension, presence or absence of inflammatory mass/abscess and free gas was not mentioned in the sonographic reports of any of the patients. Of note, none of the patients had CT scan or plain radiography as part of evaluation for typhlitis.

*Outcomes:* Eight of 12 (66.7%) patients were alive at time of completion of this research. The rest had passed away from complications not related to typhlitis.

## Discussion

This study described the clinical and imaging characteristics of patients diagnosed with typhlitis. Typhlitis was defined by the triad of fever, neutropenia, abdominal pain and radiologic evidence of inflammation of the colonic wall or the presence of 2 of 3 findings in the presence of radiologic evidence of colonic inflammation.

There had been several studies conducted concerning the incidence of typhlitis in pediatric cancer patients, albeit most of them focused on patients with acute leukemias. Incidences ranging from as low as 0.3% (6) to as high as 24% (8,) had been reported. However, most studies had shown incidences in the 2–10% range (4,9,10,12) and our calculated prevalence (4.2%) was well within the reported range. These wide range of incidences could be attributed to the different study designs used with higher incidences being reported in those using autopsy as their gold standard (8,9).

In terms of chemotherapeutic drugs, the multidrug nature of treatment regimens had made it difficult to definitively establish a causal role for individual drugs although regimens consisting of steroids, vincristine, anthracyclines (doxorubicin, daunorubicin etc.) and cytosine arabinoside had been implicated for their ability to cause bowel hypomotility and mucosal alterations that predispose to the development of typhlitis (1,2,6,10). Our study found that vincristine was part of the chemotherapy regimen in the majority of patients with typhlitis and cytosine arabinoside was used in half. Intrathecal methotrexate was part of the treatment regimen in two-third of our cases. Vincristine's association with typhlitis in our study could be attributed to the ubiquitous use of the drug in the treatment of the majority of hematologic malignancies. Doxorubicin and daunorubicin (anthracycline drugs) were used in less than 50% of the cases. Use of steroid as part of chemotherapy was recorded in 83.3% of patients.

Although typhiltis is usually a clinical diagnosis, the role of imaging, in confirming the presence of the disease and subsequent follow up, is well established (1,2,3). Our study had shown that ultrasound was able to provide all the relevant information to allow proper diagnosis and management. It also has several advantages: ultrasound is portable, radiation free, and less expensive with regards to equipment and room preparation, with no need for transporting and mixing immunosuppressed patients with other patients in waiting areas or hallways and is more readily available than other imaging modalities. Moreover, ultrasound's diagnostic yield is better than plain radiography, and fluoroscopy and is as good as CT, which in other settings can be the modality of choice.

With respect to outcomes and complications, none of the patients in our study died of typhlitis. This was found to be inconsistent with the high mortality rates seen in other low to middle income countries which reported mortality rates ranging between 24 and 45% (11,12)

Due to the retrospective nature of the study, the full data set of patients needed to obtain a complete picture of disease status in our hospital could not be achieved. A significant limitation encountered was the missing of medical records of more than half of the patients admitted during the study period. In addition, incomplete documentation of patients' clinical data and inconsistent reporting of imaging findings were encountered. Future studies with larger, preferably multi-institutional samples can provide standardized clinical and imaging protocols for better care. Hence, we believe a broader multidisciplinary prospective study approach could lead to a better understanding of this under-studied entity. In conclusion, typhlitis is an uncommon but expected complication in children undergoing treatment for malignancies, and more commonly seen in those with hematologic malignancies (acute leukemias, lymphomas). Our experience supports the affordable use of sonography for diagnosis and follow up allowing portable technique, proper isolation of immunosuppressed patients and avoiding radiation, in a resource-limited setting such as ours.
